# Academic performance in moderately and late preterm children in the United States: are they catching up?

**DOI:** 10.1038/s41372-024-01938-y

**Published:** 2024-03-18

**Authors:** Nicole E. Lock, Mark D. DeBoer, Rebecca J. Scharf, Sarah E. Miller

**Affiliations:** 1 Department of Pediatrics, Division of Neonatology, University of Virginia, Charlottesville, VA USA; 2 Department of Pediatrics, Division of Endocrinology, University of Virginia, Charlottesville, VA USA; 3 Department of Pediatrics, Division of Developmental Pediatrics, University of Virginia, Charlottesville, VA USA

**Keywords:** Outcomes research, Paediatrics

## Abstract

**Objective:**

To determine whether preterm birth of 32–36 6/7 weeks gestation affected school performance from kindergarten through fifth grade.

**Study design:**

We assessed 14350 term infants and 1195 32–36 6/7 weeks gestation infants followed in the Early Childhood Longitudinal Study Kindergarten 2011 cohort for classroom performance in kindergarten-fifth grade. Multivariable regression was performed for comparisons, and data were weighted to be representative of the US population.

**Results:**

Children born 35–36 6/7 weeks gestation had no significant difference in their academic scores or performance, while 32–34 6/7 weeks’ children had lower academic scores and teacher performance scores when compared to term children. Children born between 32 and 36 6/7 weeks gestation had higher odds of individualized education plan needs and had learning disability diagnoses compared to term children.

**Conclusions:**

Children born between 32 and 34 6/7 weeks gestation have poor school performance compared to term children. Children born between 32 and 36 6/7 weeks gestation are at risk for learning disabilities and likely benefit from continued support and services to improve achievement throughout school.

## Introduction

In the United States, preterm birth, which is defined as less than 37 completed weeks of gestation, occurs in 10.5% of all births, which has ranged from 10–10.5% of all births from 2019–2022 [1]. The largest number of preterm births occur from 32–36 6/7 weeks gestation, comprising approximately 84% of preterm births [1]. Preterm birth is associated with increased morbidity and mortality, and preterm infants even at 35–36 weeks’ have more medical problems compared to term infants [2–4].

Studies on neonatal outcomes have historically focused on the extremely premature or low birth weight infants, and include mostly NICU-based populations in these studies [5, 6]. While extremely premature infants are closely followed after NICU discharge, many moderately and late preterm children are not closely followed by developmental specialists [2, 7]. Preterm infants have previously been shown to be at risk for poor school readiness, which can lead to worse school outcomes [8, 9]. Previous regional studies have shown that moderately and late preterm children have lower performance in mathematics and language arts and have higher rates of learning difficulties [10]. This population of children also have increased special education support needs and attention and behavior complications [11, 12]. Recent meta-analysis on children born preterm demonstrated significantly lower mathematics, reading, and spelling scores compared to term infants and showed increased special education assistance [13]. Focus has shifted to include the late preterm infants and their neurodevelopmental and school outcomes, showing poor overall achievement and decreased cognitive assessment compared to term children [14–16]. While these studies are widespread, many are small, regional, older, and only represent a short span of education for children. Therefore, few studies were able to conclude whether these relationships were present after adjusting for sociodemographic factors and whether these differences persist over time. Moreover, there have not been any recent longitudinal US studies in the era of maternal corticosteroid treatment for women at risk of preterm birth.

Our study objective was to evaluate the elementary school outcomes and performance of children born between 32 and 36 6/7 weeks gestation compared to children born after 37 weeks’ in the United States. Our hypothesis was that the children born between 32 and 36 6/7 weeks gestation would have greater rates of learning difficulties and lower achievement compared to term children. This study has relevance for identifying children at risk who could benefit from intervention to improve school outcomes.

## Materials and methods

All data were obtained from the Early Childhood Longitudinal Study, Kindergarten Class of 2010-2011 (ECLS-K:2011). The ECLS-K:2011 is a voluntary, nationally representative, longitudinal cohort study consisting of 18,174 children sampled across the United States upon entry into kindergarten during the 2010–2011 school year [17]. The children came from diverse socioeconomic, racial, and ethnic backgrounds. The study was conducted by the National Center for Education Statistics (NCES) within the Institute of Education Sciences of the U.S. Department of Education to examine school readiness, grade school experiences, and child development. The NCES ethics review board approved the study. Parental consent was obtained for all children participating by the ECLS study group. The study included direct child assessments, teacher surveys, and parental surveys. Evaluations were initially performed during the fall of kindergarten, with subsequent follow-up evaluations performed every year through fifth grade. Direct child assessments were performed by trained field staff from the NCES at the children’s respective schools.

The child’s gestational age was obtained from parental surveys, as were demographic data including race and ethnicity, household income level, and parental education. The gestational age of the child was calculated based on survey responses to the question of how many days or weeks early the child was born. Children who were not identified as early were considered to be born term and assigned a gestational age of 40 weeks. Infants were divided into the following gestational age categories based on the WHO classification: term (≥37 weeks’) and preterm (<37 weeks’) [18]. The preterm category was further divided into the following categories: 35 0/7–36 6/7 weeks’, 32 0/7–34 6/7 weeks’, 28 0/7–31 6/7 weeks’, and <28 weeks’. Due to low sample numbers, the <28 to 31 6/7 weeks’ infants were not included in the separate preterm gestation analysis. 35 weeks was used as the separation points for the two preterm groups because a large amount of neonatal intensive care units use 35 weeks as the cutoff for required NICU admission.

Academic test scores were determined by direct cognitive assessments in reading, mathematics, and science. Assessments were individually administered, two stage adaptive tests, where assessors asked the children different questions related to images that were presented to the children. The first stage included items covering a broad range of difficulty, which then determined while one of three second stage tests the child was administered (low, middle, or high difficulty). The assessments were created for the ECLS studies, and were adapted from many commercial assessments with copyright permission. More information on the specific knowledge tested for each subject can be found in the ECLS user’s manual [17].

Teacher and school surveys were administered each year to report on school demographic data and specific variables about the child. Teachers were asked to evaluate the performance of each child to see if they were performing below grade level, average for grade level, or above grade level compared to their peers. Teachers also reported on individualized education plan (IEP) enrollment and learning disabilities and diagnoses on file with the school. Parental surveys also asked these questions and were used for verification of school survey answers.

All analyses were run using SAS [19]. Sample weights were used in all analyses to adjust for the effects of non-response. We used the weights provided in the ECLS-K:2011 manual to adjust for survey design. Sample weights also allowed us to use the sample of children involved in this study to produce estimates that are representative of all children throughout the USA who entered kindergarten in 2010–2011. All variables were evaluated for normality and univariate relationships. Demographic data was weighted and analyzed with pair-wise comparisons with Bonferroni correction. Multivariable linear regressions were used to analyze the relationship between gestational age and academic scores. Multivariable logistic regressions were used to analyze academic performance for children of different gestational age categories to determine the odds of performing below average compared to peers in school. Chi-square analysis was used to compare percentage of children with IEPs and diagnoses. Repeated measures data analyses were performed to determine if student testing improved over time compared to gestational age category. Confounders included in the analyses were sex, race, household income level, school type, and home setting, which have all been shown to affect rates of preterm birth and academic outcome [20, 21]. Sensitivity analysis was performed on the sample data to determine if any misclassification of gestational groups affected the outcomes of the analyses.

## Results

Of the 18 174 children enrolled in the study, 14 350 children were determined to be term gestation of at least 37 weeks’, 773 were born at 35–36 6/7 weeks gestation, and 422 were born at 32–34 6/7 weeks gestation. One hundred and twenty-two were very preterm (28–31 6/7 weeks gestation) and 35 were born extremely preterm (<28 weeks gestation). Given the small sample size, and the focus of this study, children who were born at less than 32 weeks’ were excluded from analysis. Gestational age was unable to be determined for 2 472 children as their parents did not complete the birth history section; these children were excluded from the analysis. Demographic information of the three groups of interest is shown in Table 1 with *p*-values from Bonferroni correction analysis. Notably, compared with term infants, there were more male children and more African American children in both of the preterm groups, which is similar to published data that show males and African American infants are more likely to be born preterm [22, 23]. There was less Hispanic children and less children from suburban locations in the 35-36 6/7 weeks’ sample group compared to the term children group, but it was not a significant difference. More children were sampled from the southern region of the UA in all groups. In all three groups, the most common parental education level was less than four years of college, but there was no significant difference between the three groups.

**Table 1:** Cohort demographics comparing 32–34 6/7 weeks gestation, 35–36 6/7 weeks gestation, and at least 37 weeks gestation children groups^a^.

	32–34 6/7 weeks’	*p* value	35–36 6/7 weeks’	*p* value	≥37 weeks’
Total, *n*	422		773		14350
Male, *n* (% weighted)	245 (58.0)	0.076	440 (57.0)	0.040	7303 (50.9)
Race, n (% weighted)					
White	199 (47.1)	0.728	416 (53.9)	0.329	7466 (52.0)
African American	77 (18.2)	0.001	133 (17.2)	0.013	1832 (12.8)
Hispanic	106 (25.2)	0.606	145 (18.7)	0.060	3600 (25.1)
Asian	18 (4.2)	0.990	28 (3.7)	0.990	627 (4.4)
Other (Hawaiian, American Indian, Alaskan native)	23 (5.3)	0.999	51 (6.6)	0.999	821 (5.7)
Attended public school, *n* (% weighted)	380 (90.2)	0.735	681 (88.1)	0.547	12803 (89.2)
Location, *n* (% weighted)					
City	127 (30.1)	0.999	261 (33.8)	0.518	4461 (31.1)
Suburban	139 (32.9)	0.642	214 (27.7)	0.684	4810 (33.5)
Town	46 (10.9)	0.771	70 (9.1)	0.656	1590 (11.1)
Rural	102 (24.1)	0.411	200 (25.9)	0.119	3193 (22.3)
Region, *n* (% weighted)					
Northeast	72 (17.0)	0.999	132 (17.1)	0.148	2277 (15.9)
Midwest	79 (18.6)	0.352	146 (18.9)	0.196	3212 (22.4)
South	197 (46.7)	0.471	340 (44.0)	0.556	5348 (37.3)
West	75 (17.7)	0.677	154 (20.0)	0.999	3509 (24.5)
Highest parental education level, *n* (% weighted)					
No high school degree	30 (7.1)	0.295	48 (6.2)	0.282	1284 (9.0)
High school diploma	76 (18.1)	0.686	145 (18.8)	0.843	3018 (21.0)
<4 years of college	157 (37.2)	0.403	304 (39.3)	0.570	4802 (33.5)
College degree	100 (23.7)	0.999	177 (22.8)	0.999	3296 (23.0)
Post graduate degree	58 (13.9)	0.999	100 (12.9)	0.999	1901 (13.3)
Income level of household, *n* (% weighted)					
<$20,000	53 (12.5)	0.484	117 (15.1)	0.999	2343 (16.3)
$20,000–40,000	125 (29.5)	0.499	154 (19.9)	0.692	3001 (20.9)
$40,000–65,000	48 (11.4)	0.953	107 (13.8)	0.348	2221 (15.5)
$65,000–100,000	70 (16.7)	0.271	156 (20.1)	0.484	2560 (17.8)
>$100,000	73 (17.3)	0.126	132 (17.1)	0.999	2368 (16.5)

^a^Sample numbers were weighted per the Early Childhood Longitudinal Study. P-values calculated through Bonferroni correction analysis to compare to the at least 37 weeks gestation group.

**Fig. 1:**
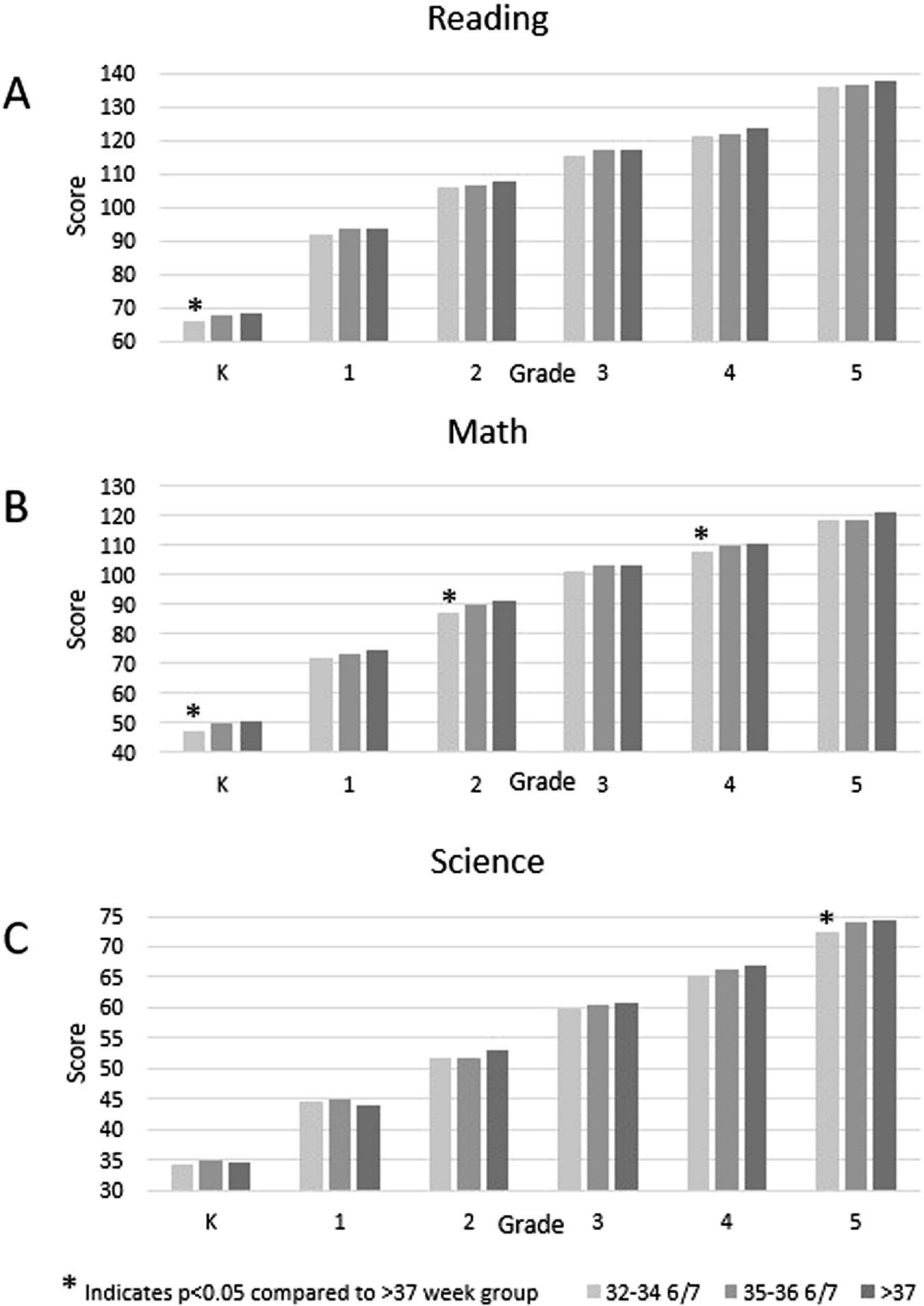
Adjusted mean test scores. Mean test scores for 32–34 6/7 weeks gestation children and 35–36 6/7 weeks gestation children compared to children at least 37 weeks gestation from kindergarten through fifth grade in **A** Reading, **B** Mathematics, and **C** Science. Asterisk indicates statistical significance of *p* < 0.05. Analyses were adjusted for gender, race, household income level, school type, and home setting. K stands for Kindergarten.

When comparing the academic scores in reading, mathematics, and science from the direct child assessments, children born 35–36 6/7 week had no significant difference from term children in reading, mathematics, or science (Fig. 1). Children born 32–34 6/7 weeks’ had significantly lower scores compared to term children for reading in kindergarten, for science in fifth grade, and for mathematics in kindergarten, second grade, and fourth grade. When comparing individuals over a longitudinal analysis over the six years of testing, gestational age was found to be a significant effector on the model, with a *p* value of <0.0001 (Supplementary Table 1).

**Fig. 2:**
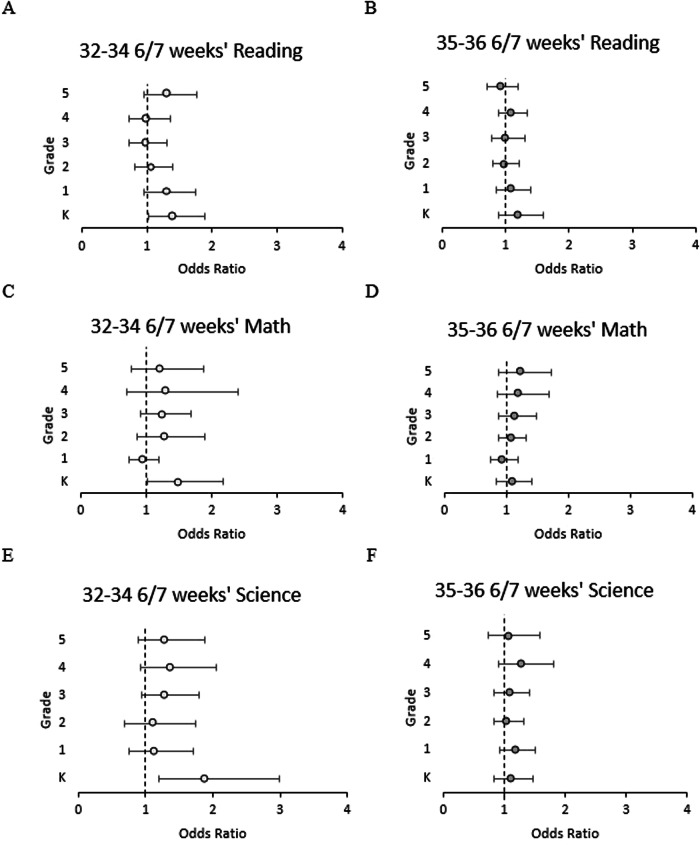
Adjusted odds ratio for below average subject performance. Odds ratio for student to have below average performance as rated by the teacher from kindergarten through fifth grade in subjects: reading for **A** 32–34 6/7 weeks gestation children and **B** 35–36 6/7 weeks gestation children; mathematics for **C** 32–34 6/7 weeks gestation children and **D** 35–36 6/7 weeks gestation children; and science for **E** 32–34 6/7 weeks gestation children and **F** 35–36 6/7 weeks gestation children. Higher number indicates worse performance. Analyses were adjusted for gender, race, household income level, school type, and home setting. K stands for Kindergarten.

Teacher assessments were completed for each child in the study to describe if they were below, equal to, or above the average performance for grade level. When comparing children born 32–34 6/7 weeks’ to term children, 32–34 6/7 weeks’ children were more likely to perform significantly below grade average in reading, mathematics, and science in kindergarten with adjusted odds ratios of 1.40 (95th percent confidence interval CI 1.03, 1.89), 1.49 (CI 1.02, 2.17), and 1.89 (CI 1.20, 2.99), respectively (Fig. 2). Significance was lost after kindergarten in each subject. For 35-36 6/7 weeks’ children compared to term children, different trends emerged. In reading, these children appeared to improve as they progressed in grade level; their odds of performing below grade level dropped from 1.19 (CI 0.89, 1.60) to 0.92 (CI 0.71, 1.19) from kindergarten to fifth grade, respectively. In mathematics, 35-36 6/7 weeks’ children were at higher odds of being below grade level performance as grade level increased, from 1.09 (CI 0.84, 1.42) to 1.23 (CI 0.87, 1.73). Science performance for 35–36 6/7 weeks’ children did not exhibit a clear trend, with an adjusted odds ratio for being below grade level that ranged from 1.05 (CI 0.83, 1.32) to 1.29 (CI 0.91, 1.81) between grades.

**Fig. 3:**
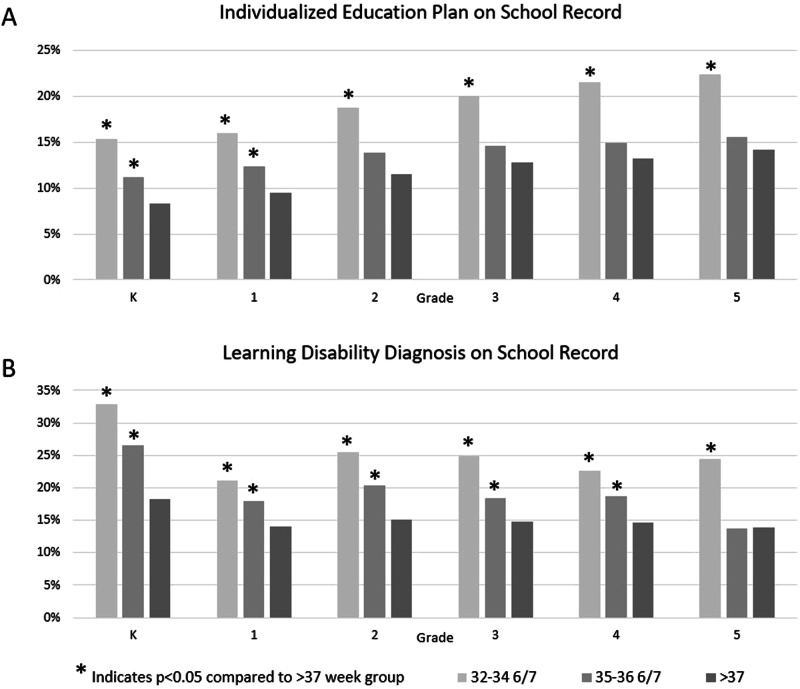
Unadjusted IEP and learning disability diagnoses percentages. **A** Unadjusted percentage of children with individualized education plan (IEP) on file with the school and **B** Unadjusted percentage of children with a learning disability on file with the school. Learning disability included a diagnosis of speech impairment, intellectual disability, visual impairment, hearing impairment, autism, or developmental delay. Asterisk indicates statistical significance of *p* < 0.05 compared to at least 37 weeks’ children. K stands for Kindergarten.

**Fig. 4:**
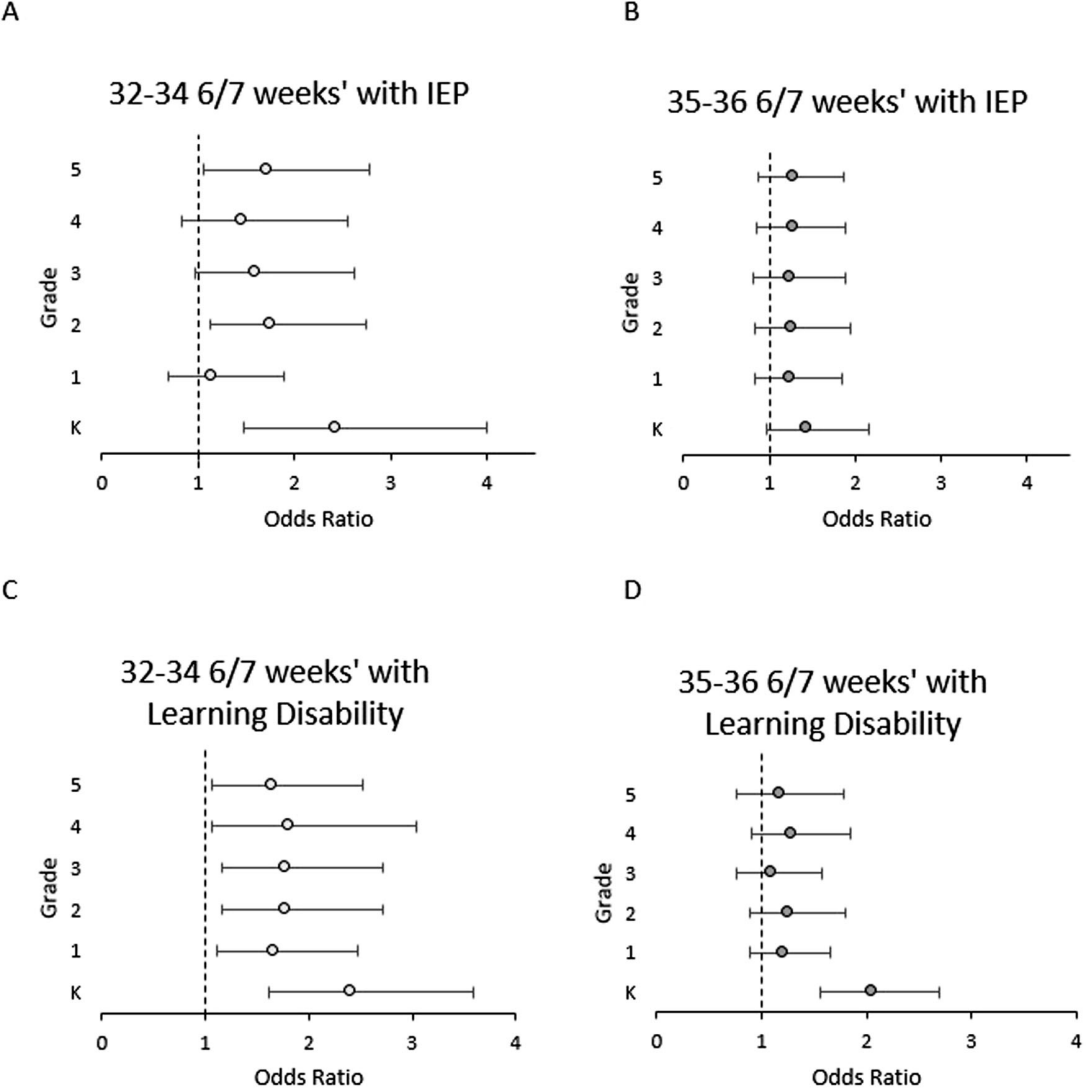
Adjusted odds ratios for IEP and learning disability diagnoses. Odds ratio for **A** 32–34 6/7 weeks gestation and **B** 35–36 6/7 weeks gestation children to have an individualized education plan (IEP) on file with the school. Odds ratio for **C** 32–34 6/7 weeks gestation and **D** 35–36 6/7 weeks gestation children to have a learning disability on file with the school. Higher number indicates higher odds. Learning disability included a diagnosis of speech impairment, intellectual disability, visual impairment, hearing impairment, autism, or developmental delay. Analyses were adjusted for gender, race, household income level, school type, and home setting. K stands for Kindergarten.

Individualized education plans (IEP) were on file for a significantly larger percentage of 35–36 6/7 weeks’ children compared to term children in kindergarten and first grade, and for every grade from kindergarten to fifth grade in 32–34 6/7 weeks’ children (Fig. 3A). For example, at first grade, 16% of 32–34 6/7 weeks’, 12.4% of 35–36 6/7 weeks’, and 9.5% of children at least 37 weeks gestation had an IEP on file, while at fifth grade these percentages had increased to 22.4%, 15.5%, and 14.2%, respectively. When comparing children born 32–34 6/7 weeks’ to term children, the adjusted odds ratio for an IEP in a 32–34 6/7 weeks’ and 35–36 6/7 weeks’ child was higher when compared to a term child (Fig. 4A), with the highest odds at kindergarten for both 32–34 6/7 weeks’ and 35–36 6/7 weeks’ children at 2.43 (CI 1.48, 4.0) and 1.44 (CI 0.96, 2.16), respectively.

Learning disabilities were also found in a statistically significantly higher proportion of children born 32–34 6/7 weeks’ and 35–36 6/7 weeks’ compared to term children in grades kindergarten through fifth grade, except for 35–36 6/7 weeks’ children in fifth grade (Fig. 3B). For example, in kindergarten, 32.9% of 32–34 6/7 weeks’ children, 26.5% of 35–36 6/7 weeks’ children, and 18.3% of term children had a learning disability diagnosis, while at fourth grade these percentages had shifted to 22.6%, 18.7%, and 14.6%, respectively. Learning disabilities included speech impairments, intellectual disability, visual impairments, hearing impairments, autism, and developmental delay. The adjusted odds ratio for a learning disability was elevated throughout kindergarten to fifth grade, with a 2.41 higher odds (CI 1.61, 3.60) in kindergarten (Fig. 4B). 32–34 6/7 weeks’ children also had the highest odds of a learning disability in kindergarten compared to term children, at 2.05 time higher odds (CI 1.56, 2.69). 32–34 6/7 weeks’ children continued to have elevated odds through fifth grade.

## Discussion

In this study, gestational age significantly affects the academic scores of the children over time, suggesting that children born between 32 and 36 6/7 weeks gestation continue to have decreased performance throughout school compared to children born at least at 37 weeks gestation. Children born between 32 and 34 6/7 weeks’ were found to have significantly lower academic scores compared to term children in multiple grades in the subjects of reading, mathematics, and science while children born between 35 and 36 6/7 weeks’ had similar score performance compared to term children in all grades. While their scores may have been similar, both children born 32 and 34 6/7 weeks’ and 35 and 36 6/7 weeks’ had higher odds of below grade average performance when compared to children born at or after 37 weeks’ in reading, mathematics, and science. The highest odds for the 32–34 6/7 weeks’ children were in kindergarten, but this appears to improve as their grade level increases for all subjects. Conversely, 35–36 6/7 weeks’ children have higher odds of below grade average performance as grade level increases in mathematics, increasing from 9 to 23% from kindergarten to fifth grade. These findings may have relevance for the importance of providing appropriate educational services and continued surveillance of learning and developmental needs for children born premature. Preterm children may need more in-depth surveillance of developmental milestones, which can be done through early intervention or other developmental follow-up clinics until schools provide educational services.

We also analyzed the data for differences between groups for whether individualized education plans or learning disabilities were on file with the school. Both of these are markers for need of services and support in school. While all the groups of children had diagnoses of learning disabilities and IEPs, significantly more were in the 32–34 6/7 weeks gestation and 35–36 6/7 weeks gestation children groups in multiple grades. The percentage of IEPs on file increased overall over time in every group, with the highest being seen in the 32–34 6/7 weeks’ group. This aligns with other studies that have shown the difficulties preterm children face in school [12, 13, 16]. While the percentage with learning disabilities slightly decreased over grades, there were significantly more in the 32–36 6/7 weeks’ groups for all grades compared to term.

While we cannot speculate on the true cause of the difference between the preterm children groups, this study highlights the concerns that children born between 32 and 36 6/7 weeks gestation, despite potentially favorable NICU courses with no prolonged illnesses or treatments, continue to have school performance difficulties. Many children have difficulties accessing resources before they start school, which may account for the higher risks in kindergarten for both preterm groups, with some drop off noted in first grade. Children can receive resources once in school, which may be closing the gap; however, as seen in this study, the gap continues to be present and a significant effector of performance. Another possibility is that 32–34 6/7 weeks gestation children have more significant difficulties that are recognized earlier, which may account for more challenges early in school that improve, while 35–36 6/7 weeks gestation children have less follow-up and more subtle challenges in school that appear later in the schooling process. Preterm children may be getting support through school to help them succeed, which is why the classroom performance is not significant in every grade. Another question brought up by this data is the discrepancy between learning disabilities and classroom performance. While preterm children may not have significantly worse performance in school subjects, they do have more learning disabilities. Are we testing the correct thing in children to help them with their learning? There may be testing or evaluation of children that is missing that should be assessed more thoroughly. Overall, closer evaluation of preterm children is necessary to help find any deficits or delays earlier, especially in resource-limited areas.

Further research is needed to help identify the children within these risk groups who would benefit from early intervention. Preterm children are at risk of attention disorders and learning disabilities, so birth history may be an important part of the evaluation process for children as they enter school [24, 25]. Pediatricians play a key role in preparing children for school, and can be a major advocate for continued support for children. State or local databases that track children may be a beneficial way to follow children from birth through elementary school, and can be a way to identify those children who are not meeting milestones and continue to need therapies through early intervention. Preschools can be a great intervention for children, and a way to improve both social and learning skills amongst peers, but more resources need to be allocated into these centers and facilities to allow more children to participate and enjoy the benefits. Pediatricians should advocate for resources for their patients and their communities to improve the wellness of children.

The strength of this study is that we used a large, nationally representative cohort which was weighted to be representative of the population. Therefore, this cohort can be generalized to the population of the USA to help understand the learning needs of these children. Longitudinal assessments were used in these children to follow them as they progressed through school. The large amount of variables and covariates also allowed for evaluation into some of the many factors that can affect the population.

The limitations of the study include the lack of complete follow-up and loss of data due to relocation prior to the fifth grade. There may also have been recall bias from parent reports. This is most significant concerning the gestational age of the child. Gestational age was calculated based on self-reported parental surveys completed potentially 5 or 6 years from delivery, which may have affected the ability to accurately place children in the correct gestational age group. Mistaken gestational age reporting would more likely bias the sample towards the null, with children who were truly preterm being classified and analyzed as full term. The sample cohort had less preterm children compared to the national average of children born in 2000–2001 (9% vs 12%), but we feel the difference is acceptable for the use of our analysis. To confirm this, we ran a sensitivity analysis to determine if the 3% discrepancy could alter our results. All analyses were re-run, with no significant changes except with teacher ratings, which were similar in magnitude and direction but had slightly wider confidence intervals, with the lower limit crossing 1.0 for reading and math in the 32–34 6/7 weeks’ group (supplemental Fig. 1). We feel this sensitivity analysis overall did not substantially change the results from the central analysis, and allow the sample cohort to continue to be representative of preterm children despite a lower sample percentage than the population.

There is little data regarding birth history or neonatal morbidities that could have affected the child’s performance in school, so these covariates could not be evaluated or adjusted. This study also excluded children who did not enroll in school or did not survive to school age, which may create a bias, as it only includes children who were well enough to attend school and complete the assessments. We acknowledge that neonatal care has changed, including the provision of antenatal corticosteroids at 34-36 weeks’ or with changes in maternal health. This may alter the results compared to previous historical studies and may make it difficult to equally compare outcomes. We also recognize that this cohort of children started elementary school over ten years ago, and education has changed over the past ten years. This cohort was before the 2020 pandemic as well, but there are plans for a 2024 cohort through the ECLS for analysis of the changes in the education of children.

In conclusion, in the United States, children that are born between 32 and 36 completed weeks gestation have poorer school performance, both in standardized testing evaluation and teacher assessment. Higher risk is seen in those who were born earlier, between 32 and 34 weeks’. While the biggest difference is seen in kindergarten, both the 32–34 6/7 weeks’ and the 32–36 6/7 weeks’ groups continue with learning difficulties and require support throughout school with individualized education plans. We highlight the need for continued resources and programs for our premature infants to help them succeed in school.

### Supplementary information

Supplemental

Supplemental figure 1

## Data Availability

MDD has obtained a restricted data license to access all data from the ECLS-K:2011 cohort due to NCES’ confidentiality legislation. Public-use ECLS-K:2011 data files and user manuals are available online through the NCES to allow for variable examination and distribution.
