# Correction: Academic performance in moderately and late preterm children in the United States: are they catching up?

**DOI:** 10.1038/s41372-024-01990-8

**Published:** 2024-05-15

**Authors:** Nicole E. Lock, Mark D. DeBoer, Rebecca J. Scharf, Sarah E. Miller

**Affiliations:** 1 Department of Pediatrics, Division of Neonatology, University of Virginia, Charlottesville, VA USA; 2 Department of Pediatrics, Division of Endocrinology, University of Virginia, Charlottesville, VA USA; 3 Department of Pediatrics, Division of Developmental Pediatrics, University of Virginia, Charlottesville, VA USA

**Keywords:** Outcomes research, Paediatrics

Correction to: *Journal of Perinatology* 10.1038/s41372-024-01938-y, published online 18 March 2024

Text for Correction:

Abstract, Study Design: We assessed 14,350 term infants and 1200 32–36 6/7 weeks gestation infants followed in the Early Childhood Longitudinal Study Kindergarten 2011 cohort for classroom performance in kindergarten-fifth grade.

Results, first paragraph: Of the 18,180 children enrolled in the study, 14,350 children were determined to be term gestation of at least 37 weeks', 780 were born at 35–36 6/7 weeks gestation, and 420 were born at 32–34 6/7 weeks gestation. 120 were very preterm (28–31 6/7 weeks gestation) and 30 were born extremely preterm (<28 weeks gestation). Given the small sample size, and the focus of this study, children who were born at less than 32 weeks' were excluded from analysis. Gestational age was unable to be determined for 2480 children as their parents did not complete the birth history section; these children were excluded from the analysis.

**Table 1 Correction:**

Table 1. Cohort demographics^a,b^.

**Table:** 

		32–34 6/7 weeks'	*p* value	35–36 6/7 weeks'	*p* value	≥37 weeks'
Total, *n*		420		780		14,350
Male, *n* (% weighted)		245 (58.0)	0.076	440 (57.0)	0.040	7303 (50.9)
Race, *n* (% weighted)						
	White	199 (47.1)	0.728	416 (53.9)	0.329	7466 (52.0)
	African American	77 (18.2)	0.001	133 (17.2)	0.013	1832 (12.8)
	Hispanic	106 (25.2)	0.606	145 (18.7)	0.060	3600 (25.1)
	Asian	18 (4.2)	0.990	28 (3.7)	0.990	627 (4.4)
	Other (Hawaiian, American Indian, Alaskan native)	23 (5.3)	0.999	51 (6.6)	0.999	821 (5.7)
Attended public school, *n* (% weighted)		380 (90.2)	0.735	681 (88.1)	0.547	12803 (89.2)
Location, *n* (% weighted)						
	City	127 (30.1)	0.999	261 (33.8)	0.518	4461 (31.1)
	Suburban	139 (32.9)	0.642	214 (27.7)	0.684	4810 (33.5)
	Town	46 (10.9)	0.771	70 (9.1)	0.656	1590 (11.1)
	Rural	102 (24.1)	0.411	200 (25.9)	0.119	3193 (22.3)
Region, *n* (% weighted)						
	Northeast	72 (17.0)	0.999	132 (17.1)	0.148	2277 (15.9)
	Midwest	79 (18.6)	0.352	146 (18.9)	0.196	3212 (22.4)
	South	197 (46.7)	0.471	340 (44.0)	0.556	5348 (37.3)
	West	75 (17.7)	0.677	154 (20.0)	0.999	3509 (24.5)
Highest parental education level, *n* (% weighted)						
	No high school degree	30 (7.1)	0.295	48 (6.2)	0.282	1284 (9.0)
	High school diploma	76 (18.1)	0.686	145 (18.8)	0.843	3018 (21.0)
	<4 years of college	157 (37.2)	0.403	304 (39.3)	0.570	4802 (33.5)
	College degree	100 (23.7)	0.999	177 (22.8)	0.999	3296 (23.0)
	Post graduate degree	58 (13.9)	0.999	100 (12.9)	0.999	1901 (13.3)
Income level of household, *n* (% weighted)						
	<$20,000	53 (12.5)	0.484	117 (15.1)	0.999	2343 (16.3)
	$20,000–40,000	125 (29.5)	0.499	154 (19.9)	0.692	3001 (20.9)
	$40,000–65,000	48 (11.4)	0.953	107 (13.8)	0.348	2221 (15.5)
	$65,000–100,000	70 (16.7)	0.271	156 (20.1)	0.484	2560 (17.8)
	>$100,000	73 (17.3)	0.126	132 (17.1)	0.999	2368 (16.5)

^a^*p*-values based on Bonferroni correction analysis comparing preterm groups (32–34 6/7 and 35–36 6/7 weeks’) to the term group (≥37 weeks’).

^b^SOURCE: U.S. Department of Education, National Center for Education Statistics, Early Childhood Longitudinal Survey, Kindergarten, 2011.

