# Supplementary material for: Academic performance in moderately and late preterm children in the United States: are they catching up?
Source: J Perinatol. 2024 Mar 18;44(6):819–26. doi: 10.1038/s41372-024-01938-y (PMC11161401; doi:10.1038/s41372-024-01938-y)
Supplement: Supplementary file 1 — Supplemental [file 41372_2024_1938_MOESM1_ESM.docx]

Supplemental table:

Supplemental table 1.

| Supplemental Table 1. Longitudinal analysis of subject score performance related to gestational age | | | |
| --- | --- | --- | --- |
| **Subject** | **Beta Estimate** | **Standard error** | **P value** |
| Reading | 100.70 | 1.3922 | <.0001 |
| Math | 80.1216 | 1.4856 | <.0001 |
| Science | 50.8508 | 0.9176 | <.0001 |

Supplemental Legends:

Supplemental Table 1. Longitudinal analysis of adjusted subject score performance in relation to gestational age.

Supplemental Figure 1. Sensitivity analysis results for 32-34 6/7 weeks’ kindergarten teacher ratings for the odds of below average performance. Children were compared to term counterparts (≥ 37 weeks’) in science, math, and reading. Higher number indicates worse performance. Analyses were adjusted for gender, race, household income level, school type, and home setting as in the original analysis.
