# Supplementary figures and images for: Academic performance in moderately and late preterm children in the United States: are they catching up?
Source: J Perinatol. 2024 Mar 18;44(6):819–26. doi: 10.1038/s41372-024-01938-y (PMC11161401; doi:10.1038/s41372-024-01938-y)

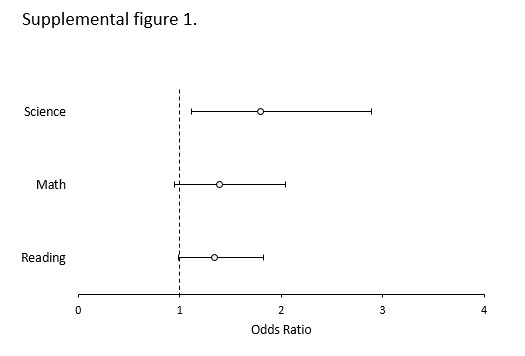

Supplement: Supplementary file 2 — Supplemental figure 1 [file 41372_2024_1938_MOESM2_ESM.jpg]
